# An inactivated novel chimeric FAdV-4 containing fiber of FAdV-8b provides full protection against hepatitis-hydropericardium syndrome and inclusion body hepatitis

**DOI:** 10.1186/s13567-022-01093-2

**Published:** 2022-09-30

**Authors:** Baiyu Wang, Mingzhen Song, Congcong Song, Shiyi Zhao, Panpan Yang, Qilong Qiao, Yanfang Cong, Yanling Wang, Zeng Wang, Jun Zhao

**Affiliations:** 1 College of Veterinary Medicine, Henan Agricultural University, Zhengzhou, 450046 China; 2 National Animal Health Products for Engineering Technology Research Center, Qingdao, 266111 China

**Keywords:** Fowl adenovirus serotype 4, fowl adenovirus serotype 8b, hepatitis-hydropericardium syndrome, inclusion body hepatitis, bivalent vaccine

## Abstract

Fowl adenovirus serotype 4 (FAdV-4) and FAdV-8b are causative agents of hepatitis-hydropericardium syndrome (HHS) and inclusion body hepatitis (IBH), respectively. HHS and IBH co-infections were often reported in clinical, yet there are no commercially available bivalent vaccines for prevention and control of both FAdV-4 and -8b. In the present study, a chimeric FAdV-4 was firstly generated by substituting fiber-1 of FAdV-4 with fiber of FAdV-8b. The chimeric virus, rFAdV-4-fiber/8b, exhibited similar replication ability in vitro and pathogenicity in vivo to the parental wild type FAdV-4. A single dosage of vaccination with the inactivated rFAdV-4-fiber/8b induced high antibody titers against fiber-2 of FAdV-4 and fiber of FAdV-8b and provided full protection against FAdV-4 and -8b challenge. These results demonstrated that fiber of FAdV-8b could replace the role of fiber-1 of FAdV-4 in the process of viral infection, and rFAdV-4-fiber/8b could be used to make a potential bivalent vaccine for the control and prevention of HHS and IBH.

## Introduction

Fowl adenoviruses (FAdVs) are non-enveloped double-stranded DNA viruses belonging to the genus *Aviadenovirus* under the family *Adenoviridae* [1]. FAdVs are categorized into five species (FAdV-A to E) and 12 serotypes (FAdV-1 to -8a and FAdV-8b to -11) based on serum cross-neutralization test and molecular characterization [2–6]. Hepatitis-hydropericardium syndrome (HHS) and inclusion body hepatitis (IBH) induced by fowl adenovirus serotype 4 of species C and 8b of species E are economically important diseases affecting poultry industry of different continents including Asia, Europe, Africa, Australia and North America [7–14]. A recent epidemiological survey indicated that FAdV-4 and FAdV-8b accounted for 38.24% and 11.58% of 9613 suspected FAdV-infected samples collected from 28 provinces in China from 2015 to 2021 [12]. HHS is associated with up to 80% mortality and characterized by the presence of clear straw-colored effusion in the pericardial sac, enlarged livers with petechial hemorrhages and edemas in kidneys [15, 16]. IBH is associated with 10% to 30% mortality and characterized by swollen livers with pale yellow-white discoloration, pinpoint haemorrhages and formation of eosinophilic or basophilic intranuclear inclusion bodies in hepatocytes [7, 10, 17].

Fiber, hexon and penton are the major structural proteins of FAdVs forming the viral outer capsids. FAdV-1, -4 and -10 contain two fibers, namely fiber-1 and fiber-2, whereas the rest of the FAdVs including FAdV-8b contains only one fiber [18]. Fiber protein consists of a carboxyl-terminal knob domain containing the antigenic and receptor binding sites, a thin shaft of different lengths depending on the serotype of FAdVs, and an N-terminal tail non-covalently linking to the penton base [19]. Fibers are crucial for the tissue tropism, pathogenesis and infection of FAdV. Fiber-1 of FAdV-4 was found to interact with the D2 domain of chicken coxsackie and adenovirus receptor (CAR) of Leghorn male hepatocellular (LMH) cells to initiate viral infection [20]. Fiber-2 was shown to be closely related to the pathogenesis of FAdV-4 and used as an immunogen for the prevention of FAdV-4 [21–26]. Fiber of FAdV-8b was also used for the construction of subunit vaccines, providing robust protection against IBH [27, 28].

Isolation of multiple FAdV serotypes from the same diseased bird is common, indicating a lack of cross-protection among different FAdV serotypes [29–33]. Mixed infection of HHS and IBH was observed in the field [12]. However, currently there is no commercially available vaccine for the prevention of both FAdV-4 and -8b infections. FAdVs of different species possess large structural and genomic discrepancies. Previous studies have demonstrated that the virulence of FAdV-4 is independent of fiber-1, and fiber-1 directly mediates infection of pathogenic FAdV-4 [34, 35]. In the present study, the hypothesis of replacement of fiber-1 of FAdV-4 with fiber of FAdV-8b was raised for the first time. A chimeric virus of FAdV-4 containing fiber of FAdV-8b designated rFAdV-4-fiber/8b was constructed. The chimeric virus retained similar replication ability in vitro and pathogenicity in vivo to the parental wild type FAdV-4. To our knowledge, this is the first study to investigate the role of fiber in FAdVs across different species. Moreover, inactivated rFAdV-4-fiber/8b vaccine could provide full protection against FAdV-4 and -8b infection.

## Materials and methods

### Materials and ethics statements

The virulent FAdV-4 strain CH/HNJZ/2015 (GenBank No. KU558760) and FAdV-8b strain SW2021 were used for the construction of the chimeric virus. Leghorn male hepatocellular (LMH) cells (ATCC, CRL-2117) were maintained in DME/F12 medium (Thermo Fisher Scientific, MA, USA) supplemented with 10% fetal bone serum (FBS, AusgeneX, Australia) at 37 °C in a 5% CO_2_ incubator for rescue and in vitro characterization of the chimeric virus. The infectious clone of CH/HNJZ/2015, p15A-cm-HNJZ, was constructed as described previously [22]. The plasmid pR6K-amp-ccdB containing the ampicillin resistance gene and the *ccdB* toxin was used as a template for amplification of the counter selectable cassette *amp-ccdB*. The competent cells used in the study were *E. coli.* strain GBred-gyrA462 which is resistant to the *ccdB* toxin and contains Redαβ recombinases and *E. coli.* strain GB05-dir which is unresistant to the *ccdB* toxin [36, 37]. Rabbit anti-FAdV-4-fiber-1 and anti-FAdV-8b-fiber antibodies were prepared by immunizing rabbits with prokaryotically expressed FAdV-4 fiber-1 and FAdV-8b fiber proteins, respectively. SPF chickens were purchased from Beijing Boehringer Ingelheim Vital Biotechnology Co., Ltd.

### Construction of the recombinant plasmid

To construct a chimeric FAdV-4 expressing the fiber of FAdV-8b, the fiber-1 gene of p15A-cm-HNJZ was firstly replaced by the *amp-ccdB* cassette amplified from pR6K-amp-ccdB by PCR. The PCR reaction contained 25 μL of the PrimeSTAR Max DNA Polymerase (Takara, Beijing, China, Cat. no. R045B), 1 μL of the forward and reverse primers Fiber-1-ac-F and Fiber-1-ac-R (10 μM), 2 μL of the template pR6K-amp-ccdB (100 ng), and 21 μL of ddH_2_O. The forward and reverse primers were composed of 33 bp of homologous arms at each side of the FAdV-4 fiber-1 gene, *Pac*I restriction enzyme digestion sites, and 18 bp of specific primers for the *amp-ccdB* cassette (Table 1). The amplicon named fiber-1-amp-ccdB was retrieved by electrophoresis following gel extraction. Next, fiber-1-amp-ccdB and p15A-cm-HNJZ were co-electroporated into *E. coli* GBred-gyrA462 for linear-circular homologous recombination as described in the previous study [22]. The correct clone named p15A-cm-HNJZ-fiber-1-amp-ccdB was linearized with *Pac*I (NEB, USA, Cat. no. R0547). Next, fiber gene of FAdV-8b was amplified from the genome of FAdV-8b strain SW2021 using primers Fiber/8b-F and Fiber/8b-R and retrieved by electrophoresis following gel extraction (Table 1). The homologous recombination of linearized p15A-cm-fiber-1-amp-ccdB and FAdV-8b fiber was conducted by T4 DNA polymerase (NEB, USA, Cat. no. M0203S) according to the previously described protocol [37]. The product was electroporated into *E. coli* GB05-dir and selected by appropriate antibiotics. The correct insertion of FAdV-8b fiber was verified by sequencing and named as p15A-cm-HNJZ-fiber/8b.

**Table 1:** **Primers for construction of the chimeric virus**

Name	Sequence (5ʹ-3ʹ)
Fiber-1-ac-F	TATTTTTAACCAATATCTTCTAGGCTCCGCCAT**TTAATTAA**TTTGTTTATTTTTCTAAA
Fiber-1-ac-R	TTCGGAATGTCTTCTTTTAGGGGCCCGGAGCAT**TTAATTAA**TTTGTTCAAAAAAAAGCC
Fiber/8b-F	CGTTTATTTTTAACCAATATCTTCTAGGCTCCGCCATATGGCGACCTCGACTCCTCACG
Fiber/8b-R	CGTTTTCGGAATGTCTTCTTTTAGGGGCCCGGAGCATTCAAGGAGCGTTGGCGGTGCTT

Bold indicates restriction enzyme sites.

### Rescue of the chimeric virus

The rescue of the chimeric virus was conducted on LMH cells. The recombinant plasmid p15A-cm-HNJZ-fiber/8b was linearized with *Pme*I (NEB, USA, Cat. no. R0560) and purified by ethanol precipitation. The linearized p15A-cm-HNJZ-fiber/8b was transfected to LMH cells when cells reached 80% confluency using Lipofectamine 3000 (Thermo Fisher Scientific, MA, USA) according to the user manual. Briefly, 5 μg of the linearized plasmid was thoroughly mixed with 10 μL of P3000 and 10 μL of Lipofectamine 3000 in 100 μL Opti-MEM and incubated at room temperature for 20 min before adding to LMH cells. The transfection mixture was replaced by DME/F12 supplemented with 2% FBS 6 h post-transfection. Once typical cytopathic effects of FAdV-4 occurred, the cells were harvested by freezing and thawing 3 times and centrifuging at 10 000 rpm for 1 min. The cell supernatant containing the viral particles was collected and stored at −80 °C. The rescued chimeric virus was verified by sequencing and named as rFAdV-4-fiber/8b.

### In vitro characterization of the chimeric virus

The rescued chimeric virus rFAdV-4-fiber/8b was subjected to continuous passaging. The viral titer and growth kinetics of the 10th passage of rFAdV-4-fiber/8b were determined on LMH cells. To determine the viral titer, the virus was serially diluted from 10^–1^ to 10^–10^ with 8 replicates for each concentration and inoculated to LMH cells cultured in the 96-well plate. The viral titer was determined by the median tissue culture infective dose (TCID_50_) by the Reed–Muench method. To determine the growth kinetics of the chimeric virus, LMH cells seeded in 6-well plates were inoculated with the virus at MOI = 0.001 and harvested at 12, 24, 36, 48, 60, 72, 84 and 96 hpi for viral titer measurement as described above.

### Western blot assay

The correct expression of fiber of FAdV-8b and complete removal of fiber-1 of FAdV-4 were confirmed with Western blot assay. LMH cells infected with FAdV-4, FAdV-8b or the chimeric virus rFAdV-4-fiber/8b were lysed with NP-40 lysis buffer with 1% PMSF (Thermo Fisher Scientific, MA, USA). The lysed cells were separated by 10% SDS-PAGE and electro-transferred onto a nitrocellulose (NC) membrane. The membrane was blocked in 5% skimmed milk in PBS for 2 h at 37 °C and incubated with the primary antibody against FAdV-4 fiber-1 or FAdV-8b fiber at 1:200 in PBS for 1 h at 37 °C. After washing with Tris-buffered saline with 0.1% Tween-20 (TBST) 3 times, the membrane was incubated in HRP conjugated goat anti-rabbit IgG secondary antibody (Abcam, Cambridge, UK) at 1:3000 in PBS for 1 h at 37 °C. The membrane was washed 3 times with TBST, treated with ECL substrate (Millipore, Germany), and visualized on Amersham Imager 600 RGB scanner.

### Pathogenicity study

To examine the pathogenicity of the chimeric virus, thirty 3-week-old SPF chickens were randomly and equally divided into three groups designated as rFAdV-4-fiber/8b, FAdV-4, and the control group. Chickens in the rFAdV-4-fiber/8b and FAdV-4 groups were inoculated with 200 μL of 2 × 10^5^ TCID_50_ of the corresponding virus via the intramuscular route. Chickens in the control group were inoculated with the same volume of serum-free DME/F12 medium. All chickens were monitored daily for clinical symptoms. A post-mortem examination was carried out for the deceased chickens during the experiment and euthanized chicken at 7 dpi. Tissue samples from the heart, liver, spleen, lung, kidneys, cecal tonsils, bursa of Fabricius, duodenum, proventriculus and pancreas were collected from all chickens for viral loads measurement. Histopathological examination was performed from heart, liver, kidney and the bursa of Fabricius.

### Evaluation of protective efficacy of the inactivated rFAdV-4-fiber/8b vaccine

To evaluate the protective efficacy of the inactivated rFAdV-4-fiber/8b vaccine against FAdV-4 and FAdV-8b infection, an oil emulsion-inactivated vaccine was generated. The chimeric virus rFAdV-4-fiber/8b was concentrated to 10^6^ TCID_50_/100 μL via dialyzing against polyethylene glycol 20,000 (Solarbio, Beijing, China) using dialysis tubing (MD44, Solarbio, Beijing, China) and inactivated by mixing with formaldehyde to a final concentration of 0.2% and incubated at 37 °C for 24 h. The inactivated rFAdV-4-fiber/8b was mixed with ADJ 501 (W/O/W) adjuvant (Zhengzhou Adjuvant Biotech CO., LTD, Zhengzhou, China) at 1:1 (v/v). The final viral dose in the inactivated oil-emulsion rFAdV-4-fiber/8b vaccine was 10^6^ TCID_50_ in 200 μL per chicken.

A total of fifty 3-week-old SPF chickens were randomly assigned to five groups designated as vaccinated and FAdV-4 challenge group, vaccinated and FAdV-8b challenge group, unvaccinated and FAdV-4 challenge group, unvaccinated and FAdV-8b challenge group, and the Sham control group. The vaccinated groups were immunized with 200 μL of the inactivated rFAdV-4-fiber/8b vaccine intramuscularly. Three weeks post-immunization, chickens were challenged with either 200 μL (2 × 10^5^ TCID_50_) of the virulent FAdV-4 strain CH/HNJZ/2015 or 200 μL (2 × 10^6^ TCID_50_) of the FAdV-8b strain SW2021, correspondingly. The Sham control group was inoculated with 200 μL of serum-free DMEM/F12 medium emulsified with ADJ501 adjuvant. All chickens were monitored for 7 days.

Sera of chickens before and after immunization were collected weekly for evaluation of the immunogenicity of the vaccine by ELISA. Heart, liver, spleen, lung, kidney, cecal tonsil, bursa of Fabricius, duodenum, proventriculus and pancreas tissue samples of chickens in each group were collected from deceased chickens during the experiment and euthanized chickens at 7 dpi for viral load measurement. Cloacal swabs were collected daily from all chickens for viral shedding measurement. Histopathological examination was performed with tissue samples of the heart, liver, kidney and the bursa of Fabricius.

### Enzyme-linked immunosorbent assay (ELISA)

The immunogenicity of the inactivated rFAdV-4-fiber/8b vaccine was evaluated by ELISA. Prokaryotically expressed and purified FAdV-4 fiber-2 and FAdV-8b fiber proteins were used as coating antigens. Briefly, 96-well plates were coated with 50 ng per well of FAdV-4 fiber-2 or FAdV-8b fiber proteins at 4 °C overnight and washed 3 times with PBS containing 0.1% Tween 20 (PBST). The plate was then blocked with 5% skimmed milk in PBS for 2 h at 37 °C. After three times of washing with PBST, 1:800 diluted serum samples were added to the plate in triplicates for incubation at 37 °C for 1.5 h. HRP conjugated rabbit anti-chicken IgY antibody (Abcam, Cambridge, UK) was then added at a dilution of 1:8000 for incubation at 37 °C for 1 h. After washing with PBST, TMB substrates (Solarbio, Beijing, China) were added to each well, and the plate was incubated at 37 °C for 10 min before stopping with 2 M sulphuric acid (Solarbio, Beijing, China). The optical density (OD) was determined at 450 nm on the Multiskan GO spectrophotometer (Thermo Fisher Scientific, MA, USA).

### Viral load and viral shedding measurement

To determine the viral loads in heart, liver, spleen, lung, kidney, cecal tonsil, bursa of Fabricius, duodenum, proventriculus and pancreas samples and the viral shedding in the cloacal swab samples, total DNA was extracted from 100 mg of tissue samples or 200 μL of the cloacal swab samples with the TIANamp genomic DNA extraction kit (Tiangen, Beijing, China, Cat. no. DP304). The viral copy numbers were determined with previously established standard curve quantitative real-time PCR (qRT-PCR) methods [22]. Briefly, the ORF14 genes of FAdV-4 and FAdV-8b were cloned into the pMD18-T vector to construct standard plasmids pMD18T-ORF14/4 and pMD18T-ORF14/8b and used as indicators for the presence of the viruses. The measurement of viral copy number in organs and cloacal swab samples by qRT-PCR was carried out as previously described [22]. The number of viral genomes was expressed as log_10_ (copy number/mg).

### Histopathological examination

At necropsy, tissue samples of the heart, liver, kidneys and bursa of Fabricius were fixed in 10% formalin for histopathological analysis. After 48 h of fixation, the samples were embedded in paraffin and cut into slices for hematoxylin and eosin staining. The stained slices were placed under light microscopy for observation of microscopic lesions.

### Statistical analysis

The statistical analysis and generation of the graphs were performed in GraphPad Prism 7. The normality of the data was examined with the D’Agostino–Pearson normality test. The Student’s *t-*test was applied for comparison of viral copy numbers between different groups. *p* values less than 0.05 and 0.01 were considered statistically significant and extremely significant, respectively.

## Results

### Generation of rFAdV-4-fiber/8b and in vitro characterization

**Figure 1:**
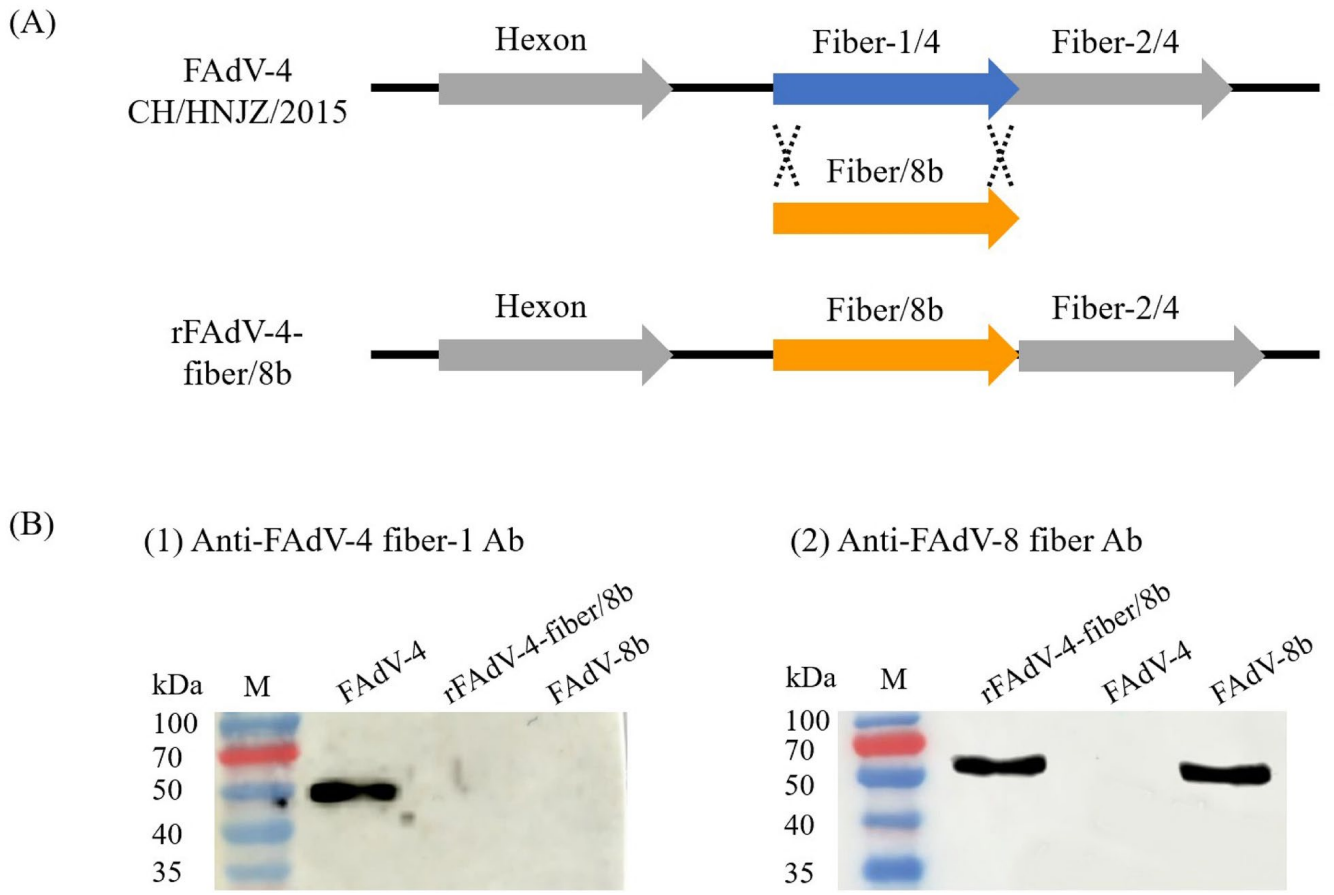
**Construction and identification of rFAdV-4-fiber/8b.** **A** Schematic illustration for the construction of rFAdV-4-fiber/8b; **B** Western blot analysis for rFAdV-4-fiber/8b. (1) The missing ~45 kDa band in the rFAdV-4-fiber/8b lane indicated the complete removal of the FAdV-4 fiber-1 in rFAdV-4-fiber/8b; (2) The ~55 kDa band indicated that FAdV-8b fiber was successfully expressed by rFAdV-4-fiber/8b.

**Figure 2:**
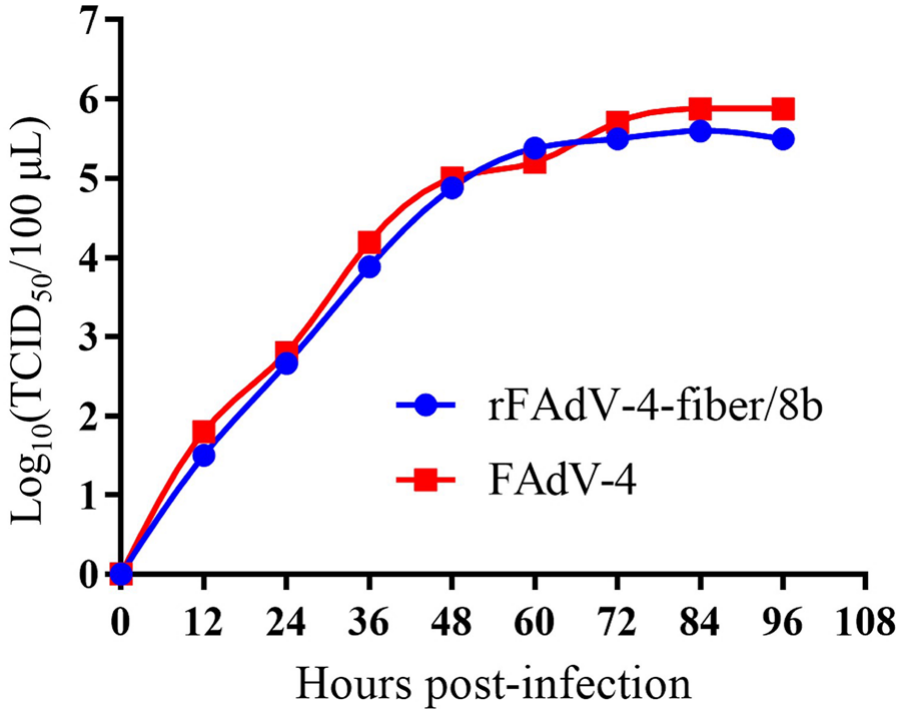
**Replication kinetics of rFAdV-4-fiber/8b and FAdV-4**. The replication kinetics of the chimeric virus rFAdV-4-fiber/8b were similar to the parental FAdV-4 in LMH cells.

A chimeric FAdV-4 with fiber-1 replaced by fiber of FAdV-8b was generated by the FAdV-4 reverse genetic platform constructed previously. Fiber-1 gene of FAdV-4 strain CH/HNJZ/2015 was substituted by the full-length fiber of FAdV-8b as indicated in Figure 1A. The overlapping region of FAdV-4 fiber-1 and fiber-2 was not affected. The chimeric virus rFAdV-4-fiber/8b was rescued after transfection of p15A-cm-HNJZ-fiber/8b to LMH cells. The successful rescue of rFAdV-4-fiber/8b was verified by viral DNA sequencing, and no gene mutation was observed in the fiber gene of FAdV-8b. The expression of the FAdV-8b fiber and the complete removal of the FAdV-4 fiber-1 were verified by Western blot (Figure 1B). The expression of FAdV-8b fiber was demonstrated by detecting the ~55 kDa band representing the FAdV-8b fiber. The ~45 kDa band representing FAdV-4 fiber-1 was not detected in the chimeric virus. The growth kinetics of the chimeric virus rFAdV-4-fiber/8b was similar to the parental virus on LMH cells (Figure 2). The peak viral titer of rFAdV-4-fiber/8b was 10^5.6^ TCID_50_/100 μL.

### rFAdV-4-fiber/8b maintains pathogenicity to susceptible chickens

**Figure 3:**
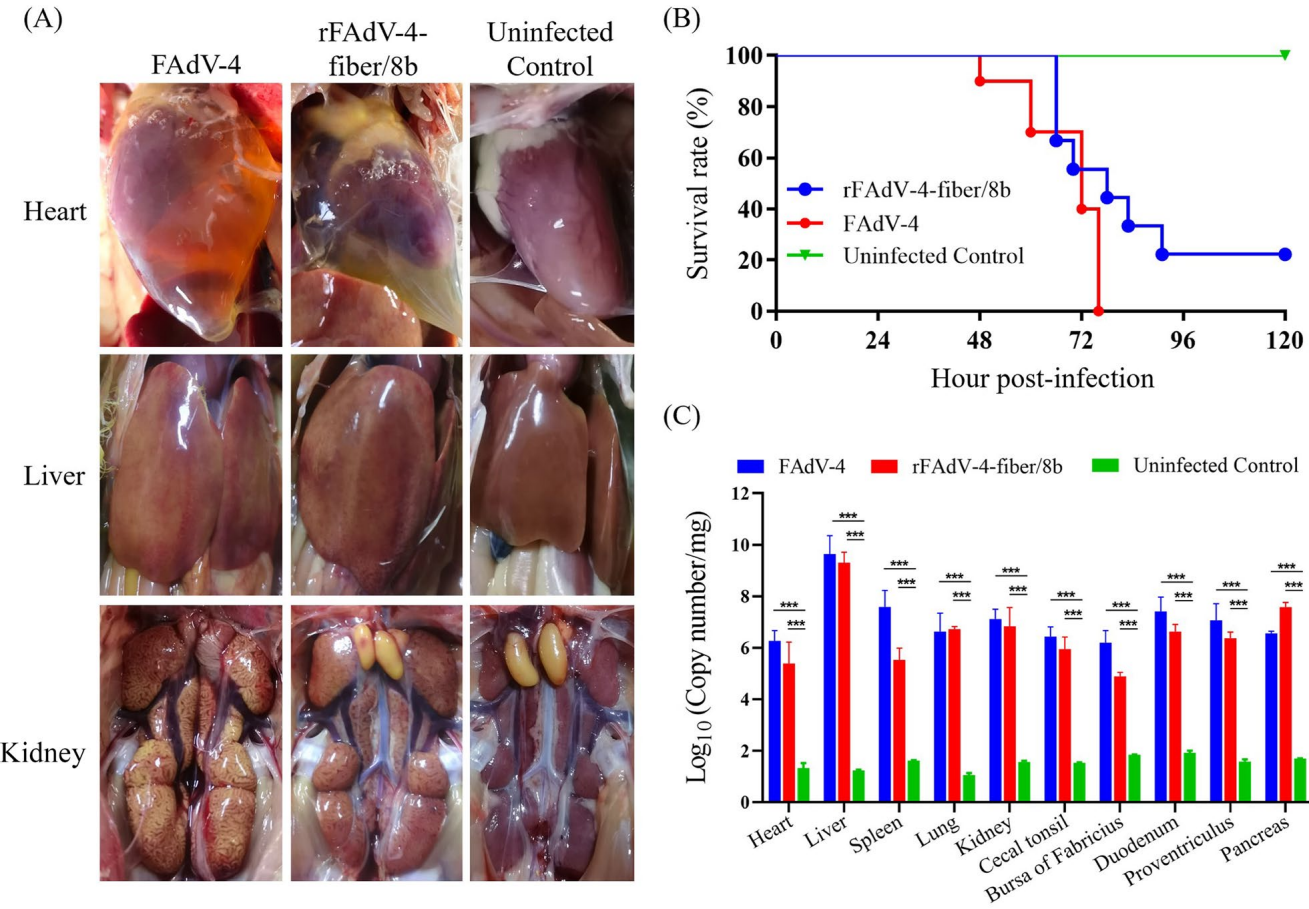
**Pathogenicity of the rFAdV-4-fiber/8b to 3-week-old SPF chickens.** **A** The representative gross lesion in the heart, liver and kidney from the chickens infected with the rFAdV-4-fiber/8b was similar to chickens infected with the parental FAdV-4. **B** The mortality of chickens infected with FAdV-4 and rFAdV-4-fiber/8b; **C** The viral loads in different organs of chickens infected with the rFAdV-4-fiber/8b and parental FAdV-4 (***p-value < 0.001).

The pathogenicity of the chimeric virus rFAdV-4-fiber/8b was examined in 3-week-old SPF chickens in comparison to the parental virus FAdV-4. Chickens infected with rFAdV-4-fiber/8b exhibited typical clinical symptoms of HHS including depression, reduced feed intake, diarrhea, and fluffy feathers. Similar to the virulent FAdV-4-infected chickens, post-mortem examination of rFAdV-4-fiber/8b-infected chickens indicated an accumulation of pericardial effusion in the pericardial sac, swollen livers and edemas in kidneys (Figure 3A). The mortality of rFAdV-4-fiber/8b-infected chickens reached 80% at 90 hpi which is slightly lower than that of the FAdV-4-infected chickens (Figure 3B). The viral loads in different organs of chickens infected with rFAdV-4-fiber/8b were at a similar level as the parental FAdV-4 and significantly higher than the control group (Figure 3C).

### Inactivated rFAdV-4-fiber/8b vaccine generated robust immune responses and provided efficient clinical protection against FAdV-4 and FAdV-8b challenge

**Figure 4:**
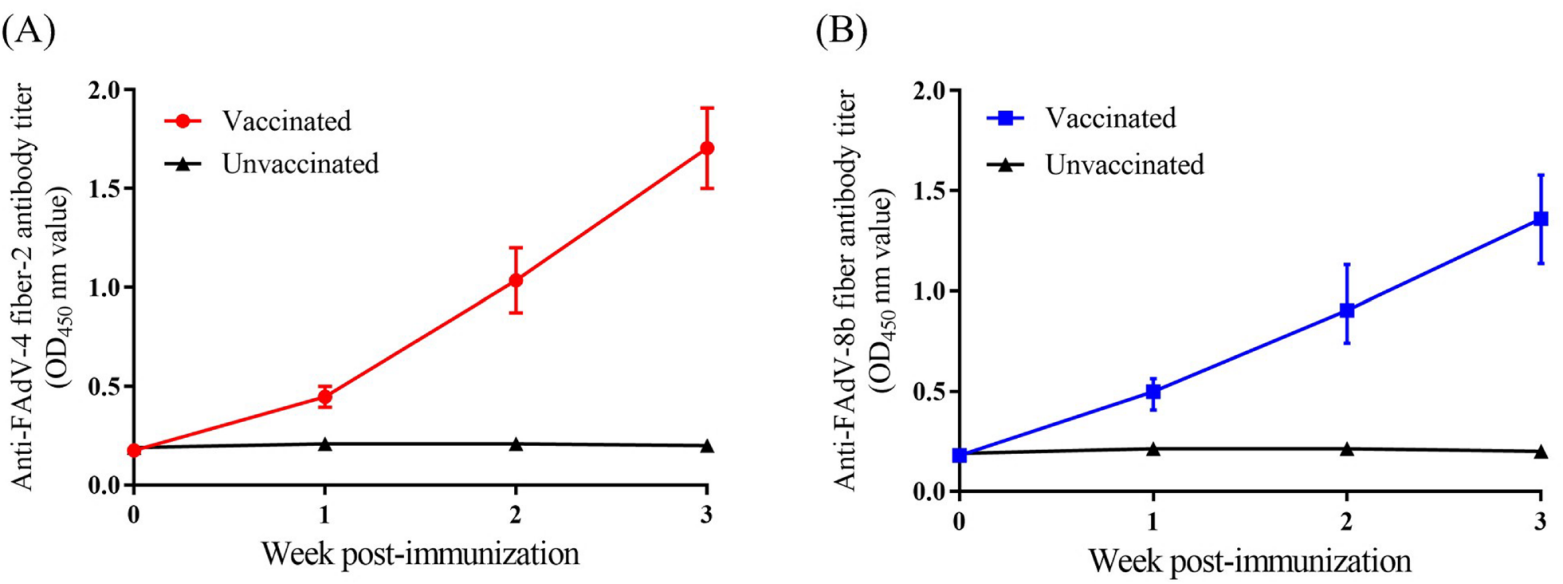
**Antibody responses against FAdV-4 fiber-2 and FAdV-8b fiber in SPF chickens immunized with the inactivated rFAdV-4-fiber/8b vaccine.** **A** The antibody responses against FAdV-4 fiber-2; **B** The antibody responses against FAdV-8b fiber.

The potential of the inactivated rFAdV-4-fiber/8b as a bivalent vaccine candidate was assessed. Three-week-old SPF chickens were immunized with one dosage of an inactivated oil-emulsion vaccine generated using rFAdV-4-fiber/8b, and the antibody responses against FAdV-4 fiber-2 and FAdV-8b fiber were determined by ELISA, respectively. For chickens immunized with the inactivated rFAdV-4-fiber/8b vaccine, the antibody responses against FAdV-4 fiber-2 and FAdV-8b fiber were elicited as early as 1 week post-vaccination and continuously increased for three weeks after vaccination (Figures 4A and B). No FAdV-4-fiber-2- and FAdV-8b-fiber-specific antibody responses were detected in unvaccinated chickens.

**Figure 5:**
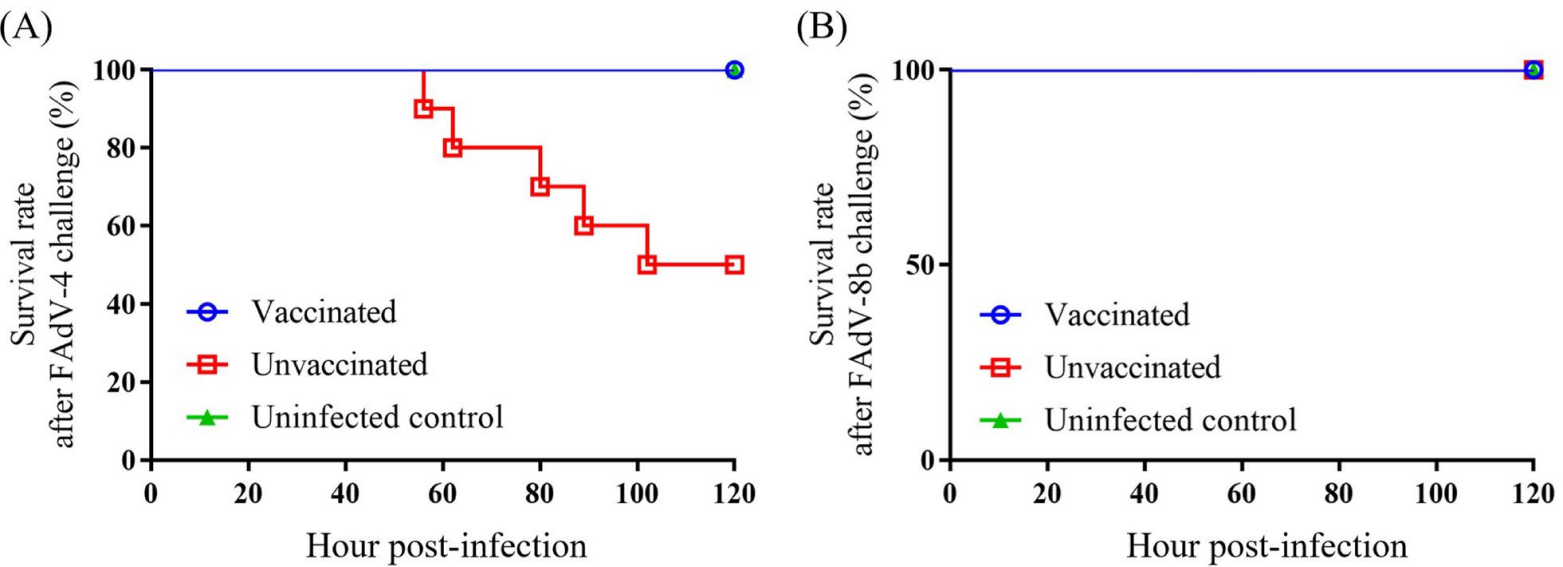
**Survival rates of chickens after challenging with virulent FAdV-4 (A) and FAdV-8b strain (B).**

Three weeks post-vaccination, both vaccinated and unvaccinated chickens in the viral challenge groups were challenged with either 2 × 10^5^ TCID_50_ of the virulent FAdV-4 strain CH/HNJZ/2015 or 2 × 10^6^ TCID_50_ of the FAdV-8b strain SW2021, correspondingly. Chickens in the unvaccinated and FAdV-4 challenge group expressed depression, decreased feed intake, diarrhea of green-yellowish watery excrement starting 2 days post-challenge. At 102 hpi, 50% of unvaccinated chickens challenged with FAdV-4 succumbed (Figure 5A), and the rest continuously experienced clinical symptoms of HHS to the end of the experiment. In contrast, all chickens immunized with the inactivated rFAdV-4-fiber/8b vaccine survived with FAdV-4 infection without any HHS-indicative clinical signs throughout the experimental period. Although both the vaccinated and unvaccinated chickens infected with FAdV-8b survived throughout the experiment (Figure 5B), the unvaccinated chickens showed depression and green watery excrement since 3 dpi.

### Inactivated rFAdV4-fiber/8b vaccine reduced histological lesions, viral loads and viral shedding

**Figure 6:**
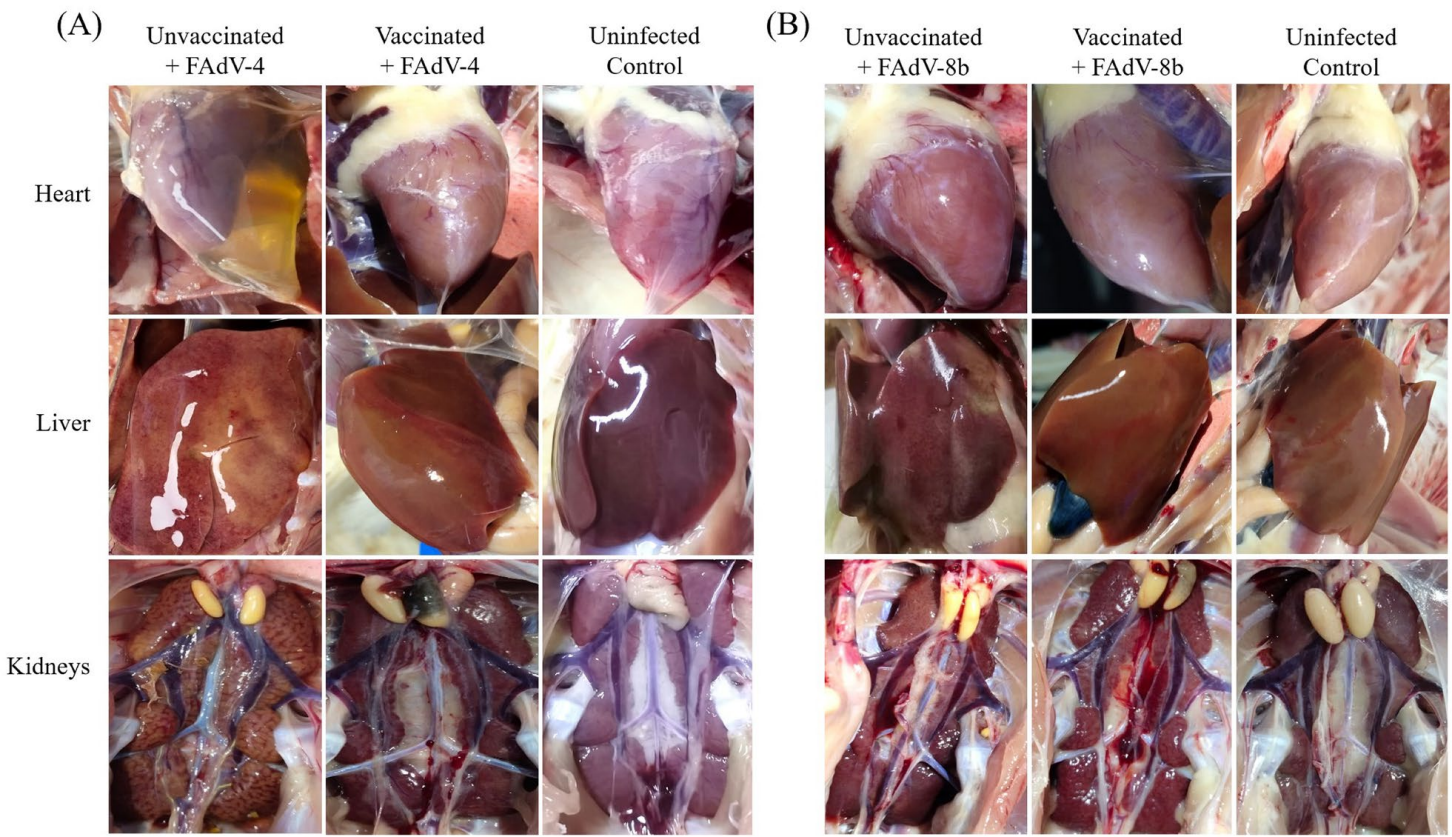
**Representative post-mortem examinations of the liver, heart and kidney from chickens challenged with virulent FAdV-4 and FAdV-8b.** **A** Accumulation of clear-yellowish effusion in the pericardial sac, swollen liver with focal haemorrhages, and edemas in the kidneys were observed in chickens from the unvaccinated and FAdV-4 challenge group. No obvious lesion was observed in chickens of the vaccinated and FAdV-4 challenge group. **B** Discoloration and pinpoint haemorrhages were observed in the liver of chickens in the unvaccinated and FAdV-8b challenge group. No obvious lesion was observed in chickens of the vaccinated and FAdV-8b challenge group.

To further assess the protective efficacy of the rFAdV4-fiber/8b inactivated vaccine, chickens were subjected to necropsy. The macroscopic lesions of the heart, liver and kidney of the unvaccinated chickens in the FAdV-4 challenge group displayed typical signs of HHS including pericardial effusion, focal necrosis on the liver, and congested kidneys, while the vaccinated chickens displayed normal healthy organs undistinguishable from the uninfected control group (Figure 6A). Similarly, unvaccinated chickens in the FAdV-8b challenge group showed swollen and friable livers with pale yellow-white discoloration and pinpoint haemorrhages, while the vaccinated chickens had no obvious liver lesions (Figure 6B).

**Figure 7:**
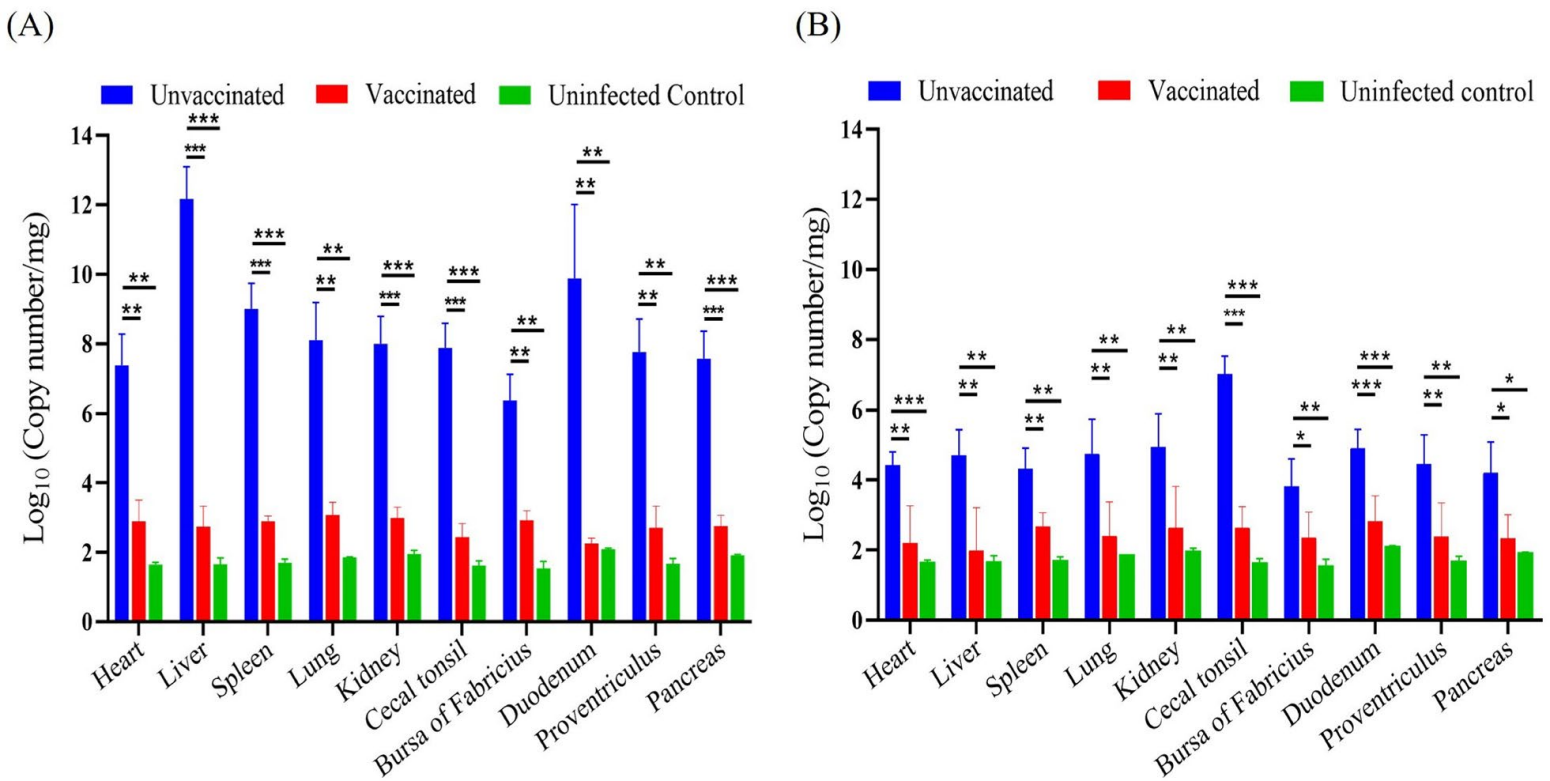
**Viral loads in different organs of chickens challenged with virulent FAdV-4 and FAdV-8b.** Viral loads in the different tissue samples were quantified by a standard curve quantitative real-time PCR method. **A** The viral loads in different tissues of chickens in the FAdV-4 challenge group. **B** The viral loads in different tissues of chickens in the FAdV-8b challenge group. In general, the viral loads in the vaccinated group and uninfected control group were significantly lower than the unvaccinated group for both viral challenge groups (*p-value < 0.05, **p-value < 0.01, ***p-value < 0.001).

The viral loads of FAdV-4 and FAdV-8b in the heart, liver, spleen, lungs, kidneys, duodenum, proventriculus, cecal tonsils, pancreas and the bursa of Fabricius of chickens were quantified by qRT-PCR. The number of viral genome copies per microgram of tissue was illustrated in Figures 7A and B. As expected, a significant decrease in viral loads was observed in chickens vaccinated with the inactivated rFAdV4-fiber/8b compared with the unvaccinated chickens, indicating the immunity generated by the inactivated rFAdV4-fiber/8b efficiently inhibited the replication of FAdV-4 and -8b in the targeting organs. High viral genome copies ranging from 10^6^ to 10^12^ and 10^4^ to 10^7^ copies per microgram of tissue were detected in the unvaccinated and FAdV-4 or -8b infected chickens, respectively, whereas only a background level of viral copy number was detected in the vaccinated chickens and uninfected chickens (Figures 7A and B).

**Figure 8:**
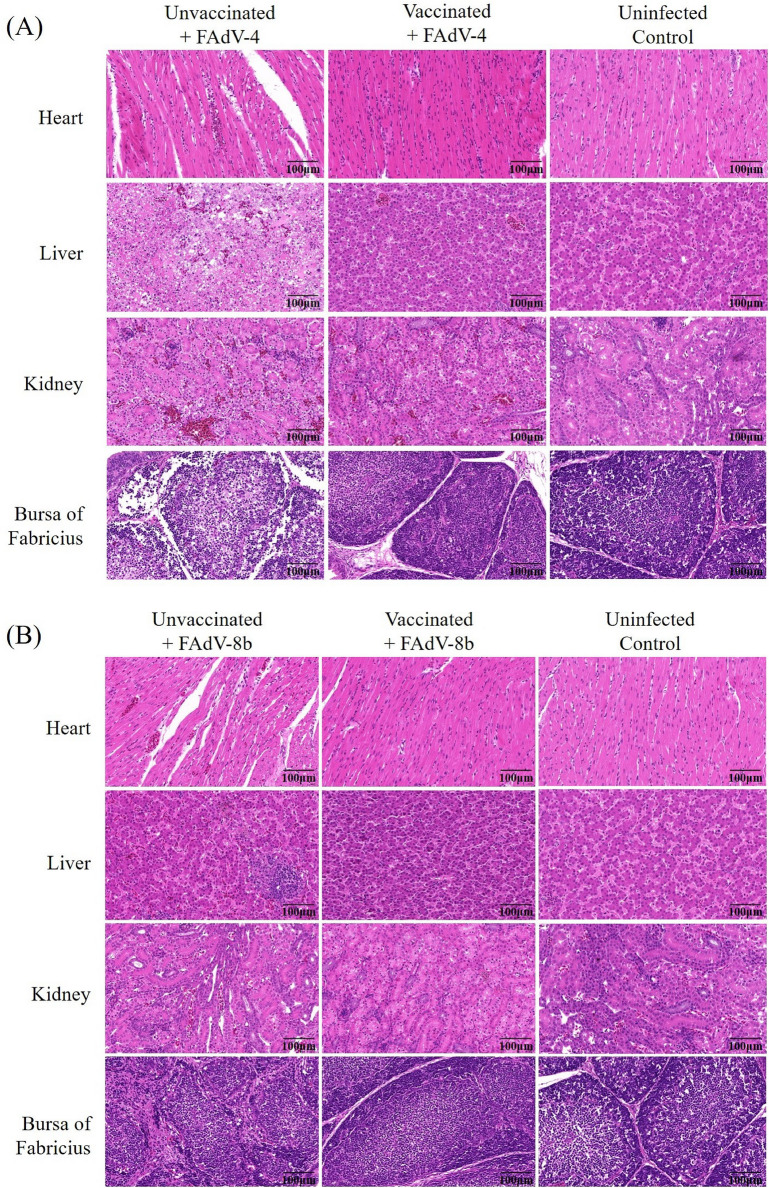
**Representative histological changes in different tissues from chickens challenged with FAdV-4 and FAdV-8b.** **A** Severe histological changes were observed in the heart, liver, kidney and the bursa of Fabricius of chickens from the unvaccinated and FAdV-4 challenge group. **B** Histopathological examination indicated inclusion bodies in the hepatocytes and disintegration of the bursa in the unvaccinated chickens challenged with FAdV-8b. No obvious histological changes were observed in the vaccinated chickens and uninfected chickens (HE staining, original magnification × 400).

The histopathological analysis of unvaccinated chickens challenged with virulent FAdV-4 revealed severe lesions, including myocardial fiber degeneration in the heart, hepatocyte necrosis with lymphocytes infiltration in the liver, hemorrhage and edemas in the kidneys and lymphoid disintegration in the medulla of the bursa of Fabricius. In contrast, the vaccinated group and the healthy control group showed no obvious pathological changes. In the case of the FAdV-8b challenge group, the unvaccinated chickens displayed hemorrhage in the heart, inclusion bodies in the hepatocytes, lymphocytes infiltration and hemorrhage in the liver, and depletion of lymphocytes in the bursa of Fabricius. The vaccinated group and the healthy control group did not show obvious abnormalities (Figure 8).

Viral shedding in cloacal swabs after FAdV-4 and FAdV-8b challenge was determined by PCR. It was observed that only two out of ten vaccinated chickens showed viral shedding on day 1 in the FAdV-8b challenge group. All chickens in the vaccinated and FAdV-8b challenge group stopped viral shedding since day 2 post-challenge. The vaccinated chickens in the FAdV-4 challenge group showed no viral shedding from day 1 until the end of the experiment (Table 2). Chickens in the unvaccinated and viral challenge groups showed continuous viral shedding throughout the experiment.

**Table 2:** **Viral shedding rate of chickens challenged with FAdV-4 or -8b in cloacal swabs**

Group	Challenged virus	Survival rate	Viral shedding (day post-challenge)				
			1	2	3	4	5
Vaccinated	FAdV-4	10/10	0/10	0/10	0/10	0/10	0/10
	FAdV-8b	10/10	2/10	0/10	0/10	0/10	0/10
Unvaccinated	FAdV-4	5/10	10/10	10/10	8/8	6/6	5/5
	FAdV-8b	10/10	8/10	9/10	9/10	9/10	10/10

## Discussion

HHS and IBH outbreaks are increasingly reported worldwide, causing numerous economic losses to the poultry industry. Since 2015, the clinical cases of HHS and IBH showed an increasing trend in China [38, 39]. Vaccination is one of the most commonly used strategies in the prevention and control of diseases. Inactivated whole FAdV-4 or subunit vaccines have been developed for the prevention of HHS [24, 40]. FAdV-8a/b fiber-based vaccines provided type-specific protection against IBH [41]. However, there are no commercial bivalent vaccines to prevent and control the spread of HHS and IBH. Bivalent vaccines for the prevention of co-infection of different FAdV serotypes were reported recently. A chimeric fiber protein, crecFib-4/11, containing epitopes from fiber-2 of FAdV-4 and fiber of FAdV-11 can provide efficient protection against FAdV-4 and -11 infection [33]. Currently, viruses inducing IBH belong mainly to species D or E, predominantly serotypes 2, 8a, 8b, and 11. As antigenically diverse fowl adenovirus types are involved in IBH in chickens, the vaccines against IBH are complicated. Schachner et al. recently reported that recombinantly expressed novel chimeras, crecFib-8a/8b and crecFib-8b/8a, containing amino acid positions 1 to 441 and 442 to 525/523 in the fibers of FAdV-8a and -8b, induced cross-neutralizing antibodies and protective responses in chickens against infections by both serotypes [28]. During the preparation of our manuscript, Lu et al. generated a recombinant FAdV-4 with fiber of FAdV-8b by inserting the fiber of FAdV-8b between fiber-1 and fiber-2 of FAdV-4 and demonstrated that the recombinant FAdV-4 could provide efficient protection against both FAdV-4 and FAdV-8b [42].

Based on previous collective data that fiber-1 directly initiated FAdV-4 infection and the virulence of pathogenic FAdV-4 is independent of fiber-1, a chimeric FAdV-4 virus, rFAdV-4-fiber/8b, with fiber-1 of the virulent FAdV-4 substituted by fiber of FAdV-8b was generated for the first time in the present study. The chimeric rFAdV-4-fiber/8b exhibited similar in vitro replication ability to the parental wild type FAdV-4 strain. The pathogenicity study of rFAdV-4-fiber/8b demonstrated that the replacement of fiber-1 of FAdV-4 with fiber of FAdV-8b did not alter the pathogenic properties of the parental FAdV-4. It is worth noting that a single dose of immunization with the inactivated rFAdV-4-fiber/8b vaccine could provide full protection against both FAdV-4 and FAdV-8b challenges. The vaccinated chickens exhibited no clinical signs throughout the experimental period, and no gross or microscopic lesions were observed after the viral challenge. The viral loads in the tested organs of vaccinated chickens were significantly lower than those in the unvaccinated chickens. Both FAdV-4 and FAdV-8b viral genome copy numbers in the tissues of chickens from the inactivated rFAdV-4-fiber/8b vaccinated group were at similar levels to those in the corresponding tissues of chickens from unchallenged control groups, and the difference did not reach statistical significance.

The initiation of viral infection is often mediated by proteins of the outer viral capsid. FAdVs are icosahedrons, and the majority of FAdV viral outer capsid is composed of hexagonal-shaped protein hexon trimers. Each vertex of the icosahedron is a penton base connected with one or two protruding fibers. High amino acid variations are found in the knob domain of fiber which result in different receptor binding sites and antigenic activities depending on different serotypes [19]. Fiber plays a crucial role in the initial recognition and interaction of FAdVs with the host. Previous research indicated FAdV-4 had a distinct infection mechanism from FAdV-1 which also contains two fiber genes. Although CAR is used as one of the cell-binding receptors for both FAdV-1 and -4, FAdV-1 binds to the host cells with the long fiber-1, whereas FAdV-4 interact with the host cells using the short fiber-1 [20]. At present, there is a lack of investigation of the infection mechanism of FAdV-8b. The substitution of fiber-1 of FAdV-4 had almost no effect on the viral infection of rFAdV-4-fiber/8b in vitro and in vivo. It is suggestive that FAdV-8b might infect the host cells in a similar way to FAdV-4, and fiber of FAdV-8b might play the same role in the initiation of infection as fiber-1 of FAdV-4. Fiber-substituted human adenovirus vectors containing foreign peptides in the adenovirus serotype 35 fiber knob have been reported [43]. The resulted fiber-substituted Ad serotype 5 vectors containing the fiber protein from Ad serotype 35 (Ad5F35) exhibit properties that render them suitable as a platform for targeted Ad vectors [44]. Zhang et al. have shown that fiber-1 modifications enable FAdV-4 to transduce human cells [45]. Our results confirmed that it was feasible to make chimeric recombinant viruses which have a broad host spectrum and can provide cross-protection against different FAdVs by modifying or replacing the fiber gene of FAdVs. This will lay a foundation for the development of gene therapy vectors and multivalent vaccine candidates.

In summary, a novel chimeric FAdV-4 with its fiber-1 substituted by fiber of FAdV-8b was generated for the first time, and this is the first demonstration of the fiber of FAdV-8b can function as fiber-1 of FAdV-4 in the initiation of FAdV-4 infection. The chimeric rFAdV-4-fiber/8b could be used to prepare a bivalent vaccine for the effective prevention and control of FAdV-4 and -8b infection. Our findings support further studies to elucidate the pathogenesis of FAdVs of different serotypes, leading to improved control strategies of HHS and IBH.

## Data Availability

The data supporting the results of this study are available from the corresponding author, Jun Zhao, upon request.
